# Disease activity and mental health symptoms in axial spondyloarthritis: concordant or discordant?

**DOI:** 10.1093/rheumatology/keaf506

**Published:** 2025-09-29

**Authors:** Sizheng Steven Zhao, Casper Webers, Elena Nikiphorou, Désirée van der Heijde, Jürgen Braun, Uta Kiltz, Sofia Ramiro, Annelies Boonen

**Affiliations:** Centre for Musculoskeletal Research, Division of Musculoskeletal and Dermatological Science, University of Manchester, Manchester, UK; NIHR Manchester Biomedical Research Centre, Manchester University NHS Foundation Trust, Manchester, UK; Care and Public Health Research Institute (Caphri), Maastricht University, Maastricht, the Netherlands; School of Inflammation and Microbial Sciences, Centre for Rheumatic Diseases, King’s College London, London, UK; Centre for Education, King’s College London, London, UK; Rheumatology Department, King’s College Hospital NHS Foundation Trust, London, UK; Leiden University Medical Centre, Leiden, the Netherlands; Rheumatologisches Versorgungszentrum RVZ Steglitz, Ruhr Universität Bochum, Berlin, Germany; Rheumazentrum Ruhrgebiet, Ruhr-University Bochum, Herne, Germany; Leiden and Zuyderland Medical Center, Leiden University Medical Center, Heerlen, the Netherlands; Care and Public Health Research Institute (Caphri), Maastricht University, Maastricht, the Netherlands; Maastricht UMC+, Dept of Rheumatology, Maastricht, the Netherlands

**Keywords:** axial spondyloarthritis, mental health, anxiety, depression, latent class analysis, group-based trajectory analysis

## Abstract

**Objective:**

We applied latent class and trajectory modelling to examine whether subgroups of axial spondylarthritis (axSpA) patients report discordant scores for disease activity and mental health symptoms at baseline and after treatment change.

**Methods:**

We analysed axSpA patients from the ASAS Health Index International Validation Study. We applied latent class analysis (LCA) using generalized structural equation modelling to identify subgroups among 1292 individuals, based on baseline Hospital Anxiety and Depression Scale (HADS) subscores and ASDAS. We applied trajectory modelling in a subset (*n* = 206) requiring treatment change, to identify subgroups of distinct trajectories for HADS and ASDAS over 6 months. All indices were standardized. Baseline characteristics were compared across identified groups.

**Results:**

For the baseline analysis, three groups were identified with concordant HADS subscores and ASDAS, with similar baseline characteristics except the high HADS/ASDAS group having more peripheral joint involvement and higher CRP levels. Trajectory analysis identified four groups with concordant HADS and ASDAS changes: 54% comparatively low baseline values, 33% medium and, of the high baseline groups, some (7%) had marked improvement (HADS-depression Δ11, HADS-anxiety Δ9, ASDAS Δ2.8), while others (6%) had limited ASDAS improvement (Δ1.4) with minimal changes in anxiety symptoms (Δ0.6).

**Conclusions:**

We did not identify the hypothesized subgroups with discordant disease activity and mental health symptoms. Instead, these domains were closely aligned at baseline and following treatment, suggesting that these symptoms influence each other. Patients with high mental health symptom burden may benefit from knowing that these symptoms often improve alongside disease activity when starting treatment.

Rheumatology key messages

- Mental health symptoms and axial spondyloarthritis disease activity (ASDAS) are generally concordant cross-sectionally and after treatment initiation.
- ASDAS and mental health symptoms are closely related, suggesting that these symptoms influence each other.
- Patients may benefit from knowing that mental health often improves with disease activity after starting treatment.

## Introduction

Axial spondyloarthritis (axSpA) is characterized by back pain that is mostly inflammatory in nature [1]. Assessment of axSpA disease activity relies entirely (e.g. BASDAI) or partly [e.g. Axial Spondyloarthritis Disease Activity Score (ASDAS)] upon subjective patient reported assessments. However, many factors can influence patients’ experience and reporting of axSpA disease activity independently of the underlying inflammatory process. Mental health comorbidities, such as anxiety and depressive symptoms, are of particular interest as they are common in axSpA (moderate-severe symptoms reported in around 15% [2]) and may influence accurate assessment of disease activity. Previous research has shown that patients with axSpA with comorbid depression report higher BASDAI and spinal pain than those without [2–4]; similarly, responses to TNF inhibitors are reduced in those with moderate-severe depressive symptoms compared with those without, whether assessed using BASDAI or ASDAS [5]. In turn, pain and functional limitation due to axial inflammation are also likely to influence mental health [6].

Understanding the inter-relationship between mental health comorbidities and reported axSpA disease activity is important, as biologic or targeted synthetic DMARDs (b/tsDMARDs) were primarily developed to treat musculoskeletal inflammation. Patients with high disease activity scores that are primarily driven by severe depressive symptoms may, in theory, not respond to b/tsDMARDs as well as those driven by high levels of axial inflammation [7]. Targeted interventions to optimize depression management may be a better approach in these subgroups. Distinguishing such ‘phenotypes’ of reported disease activity may help tailor management approaches. We hypothesize that there are groups of people with axSpA who have discordant disease activity and mental health symptoms, for example, those who have high mental health symptom burden despite low inflammatory disease activity. Our aims were to, first, apply data-driven approaches to measures of axSpA disease activity (namely the ASDAS) and mental health [subscores of the Hospital Anxiety and Depression Scale (HADS)] to identify potential groups with discordant baseline values or trajectories across these indices and, second, describe baseline patient and disease characteristics across the groups.

## Methods

This study was an ancillary analysis of the data collected for the Assessment of SpondyloArthritis international Society Health Index (ASAS HI) International Validation Study, which recruited patients with a clinical diagnosis of spondyloarthritis [8]. We included all adult (age ≥18) participants with axSpA, who fulfilled ASAS criteria for axSpA and had complete baseline ASDAS and HADS data. Those with a diagnosis of peripheral SpA were excluded. The ASAS HI Validation Study study was performed in 23 countries during 2014 and 2015. Although the study was primarily cross-sectional, recruiting centres were also asked to include at least 25% in a longitudinal arm (named ‘responsiveness’ arm in the original study). Patients in the responsiveness arm required change in therapy (initiation of NSAID, csDMARDs or TNFi) because of high disease activity and were reassessed 12–24 weeks (2–24 weeks for NSAIDs) after treatment change. All centres participating in the ASAS HI Validation Study received approval from their local ethics committee, and all participants provided written informed consent. This ancillary analysis did not require additional approvals.

### Data collection

The study collected baseline data on demographics, disease characteristics (ever had arthritis, enthesitis, dactylitis, uveitis, IBD, psoriasis; elevated CRP, HLA-B27; symptom duration, disease duration). Participants also completed a series of self-reported outcomes including axSpA-specific and non-specific indices (with higher values reflecting worse outcomes): patient global (disease activity, numerical rating scale 0–10), pain, HADS, BASDAI, BASFI. We also included perceived treatment helpfulness (from the Brief Illness Perception Questionnaire) with higher scores (scale 0–10) representing greater belief that treatments will help. ASDAS was calculated using the respective BASDAI questions, patient global and CRP. Information on treatment (NSAIDs, csDMARDs, TNFi) was also collected. For patients from the responsiveness arm, patient-reported outcomes and treatment were collected at baseline and at the reassessment visit after treatment change.

### Assessment of mental health

The HADS is a 14-item self-reported questionnaire designed to assess anxiety (HADS-A, 7 items) and depressive symptoms (HADS-D, 7 items) in non-psychiatric medical populations [9]. Each item is scored on a 0–3 scale, with total subscale scores ranging from 0 to 21. Higher scores indicate more symptom severity, with commonly used cutoffs of ≤7 as no, 8–10 mild, 11–14 moderate and ≥15 severe anxiety or depressive symptoms.

### Statistical analysis

We used a data-driven, unsupervised approach to look for the hypothesized latent (unobserved) groups that differ in baseline values or trajectories of ASDAS and HADS sub-scores. Such latent class analyses (LCA) identify categorical classes based on observed variables [10]. Latent variables are hypothetical constructs that cannot be measured directly, such as ‘phenotypes’ of inflammatory or non-inflammatory predominant ‘high axSpA disease activity’. Observed variables (in this case ASDAS and HADS) were used to model and identify these underlying classes.

Traditional clustering methods group individuals based on similarity (‘distance’ between observed variables), without assuming an underlying latent structure. Cluster analysis also forces individuals into rigid groups without providing probabilistic measures of uncertainty. By contrast, latent class analysis is a model-based approach that assumes the presence of latent subgroups in the population and assigns individuals to these latent classes based on probability distributions, accounting for measurement error and uncertainty [10]. Additionally, latent class analysis can be extended to study longitudinal trajectory [11].

In the current study, we first applied latent class analysis to identify distinct subgroups based on baseline ASDAS and HADS. Second, we applied group-based trajectory modelling to a subset who required change in therapy to identify subgroups of distinct trajectories for HADS and ASDAS over 6 months.

### Baseline latent class analysis

For the baseline analysis, we included all patients with baseline ASDAS and HADS data. We hypothesized that there would be latent groups based on concordant or discordant scores across baseline values of the three indices (ASDAS, HADS-A, HADS-D); for example, high anxiety and depressive symptoms despite low ASDAS. To identify distinct subgroups based on baseline HADS and ASDAS, LCA was performed using generalized structural equation modelling (using the gsem command in Stata). ASDAS, HADS-A and HADS-D were included simultaneously as observed measures used to define the latent classes. Because ASDAS and HADS are reported on different ranges, we standardized them to a common 0–10 scale to facilitate comparison. Models were estimated with 2–10 latent classes. The optimal number of latent classes was selected based on minimizing model-fit statistics [i.e. Bayesian and Akaike Information Criteria (BIC/AIC)] and clinical interpretability (e.g. avoiding small groups with differences that are not clinically meaningful) and then used to assign each individual a posterior probability of class membership. Each individual was assigned the class for which the posterior probability was highest. We also generated predicted probabilities for each class to visualize (as bar graphs) the difference across ASDAS, HADS-A and HADS-D.

We performed sensitivity analyses to assess whether the following changes altered the latent classes: first, we repeated models without standardizing indices; second, we included additional symptom indicators (BASDAI question on fatigue, spinal pain, BASFI); third, we repeated the analysis among only the longitudinal ‘responsiveness’ arm (used for the latent trajectory analysis below).

### Latent trajectory analysis

We also hypothesized that groups may have concordant and discordant trajectories over time after treatment; for example, ASDAS and HADS may improve in tandem or ASDAS may reduce but with persistently elevated HADS. We applied group-based trajectory modelling (using the traj command in Stata). A linear multivariate model was fitted with ASDAS, HADS-A and HADS-D simultaneously as observed variables and a time indicator variable (baseline or follow-up). All three indices were standardized to preventing differences in scale from disproportionately influencing the trajectory models. Variables were modelled using a normal distribution; potential departure from this distribution (e.g. skew) provides additional information for the latent class model and does not require additional data transformation [12]. To facilitate interpretability, we tested models with up to 10 classes or, to allow meaningful comparison of clinical and disease characteristics, when any individual classes had <5% of the overall population. The optimal number of groups was selected based on BIC/AIC and clinical interpretability. Once the final model was selected, group membership probabilities were estimated and used to assign individuals to trajectory groups.

As a sensitivity analysis, we additionally included fatigue, spinal pain and BASFI in the latent trajectory models to assess whether they influenced the trajectories of ASDAS and HADS.

For our second aim, we compared characteristics (e.g. distributions of age, sex, quality of life, physician global scores) first for the baseline latent groups and second for the latent trajectory groups.

## Results

For the baseline analysis, 1292 individuals (mean age 42 years, 68% male, 80% HLA-B27 positive) were included, among whom 72% were classified as radiographic axSpA. At baseline, the mean disease duration was 8.8 (SD 9.2) years, mean HADS-D 5.7 (SD 4.2), HADS-A 6.8 (4.3), ASDAS 2.6 (1.2) and median CRP 3.0 (IQR 2.0, 10.9) mg/l (Table 1).

**Table 1.** Characteristics of individuals included in the baseline latent class analysis

		All patients	Group 1	Group 2	Group 3	*P*-value
N		1292	633	495	164	
Age (years)		41.9 (13.2)	41.2 (13.3)	42.8 (13.4)	41.9 (11.9)	0.14
Male		884 (68%)	449 (71%)	328 (66%)	107 (65%)	0.16
Highest education attainment	Primary	114 (9%)	47 (7%)	46 (9%)	21 (13%)	<0.001
	Secondary	591 (46%)	254 (40%)	249 (51%)	88 (54%)	
	University	583 (45%)	330 (52%)	198 (40%)	55 (34%)	
Symptom duration (years)		15.2 (11.5)	14.9 (11.3)	15.7 (11.8)	15.1 (11.4)	0.54
Disease duration (years)		8.8 (9.2)	9.1 (9.0)	8.6 (9.3)	8.3 (9.6)	0.49
HLA-B27 positive		869 (80%)	462 (85%)	316 (78%)	91 (70%)	<0.001
Radiographic axSpA		924 (72%)	438 (70%)	361 (73%)	125 (77%)	0.17
Peripheral arthritis		181 (14%)	63 (10%)	82 (17%)	36 (22%)	<0.001
Dactylitis		23 (2%)	8 (1%)	9 (2%)	6 (4%)	0.027
Peripheral enthesitis		192 (15%)	70 (11%)	82 (17%)	40 (25%)	<0.001
Uveitis		47 (4%)	20 (3%)	19 (4%)	8 (5%)	0.77
Psoriasis		53 (4%)	25 (4%)	23 (5%)	5 (3%)	0.70
IBD		54 (4%)	27 (4%)	22 (5%)	5 (3%)	0.78
CRP (mg/L)		3.0 (2.0, 10.9)	2.0 (2.0, 8.2)	3.0 (2.0, 10.0)	9.0 (2.0, 30.0)	<0.001
HADS-depression (0–21)		5.7 (4.2)	2.4 (1.6)	7.6 (2.0)	13.2 (2.3)	<0.001
HADS-anxiety (0–21)		6.8 (4.3)	3.9 (2.5)	8.3 (2.8)	13.4 (3.3)	<0.001
ASDAS		2.6 (1.2)	2.1 (1.0)	2.8 (1.1)	4.0 (0.9)	<0.001
BASDAI (0–10)		4.1 (2.5)	2.8 (1.9)	4.7 (2.2)	7.2 (1.6)	<0.001
BASFI (0–10)		3.3 (2.8)	2.0 (2.2)	3.9 (2.6)	6.4 (2.4)	<0.001
ASAS-HI score (0–17), mean (SD)		6.6 (4.3)	4.0 (3.0)	8.1 (3.5)	12.3 (2.8)	<0.001
Perceived treatment helpfulness (0–10)		7.3 (2.3)	7.5 (2.3)	7.0 (2.4)	6.9 (2.3)	0.021
Predicted probability for Group 1			0.9 (0.1)	0.1 (0.1)	0.0 (0.0)	<0.001
Predicted probability for Group 2			0.1 (0.1)	0.9 (0.1)	0.1 (0.1)	<0.001
Predicted probability for Group 3			0.0 (0.0)	0.0 (0.1)	0.9 (0.1)	<0.001

Data shown as *n* (%), mean (standard deviation) or median (interquartile range). Group 1: low ASDAS/HADS; Group 2: medium ASDAS/HADS; Group 3: high ASDAS/HADS. ASAS-HI: Assessment of Spondyloarthritis International Society Health Index score; ASDAS: Axial Spondyloarthritis Disease Activity Score; BASDAI: Bath Ankylosing Spondylitis Disease Activity Index; BASFI: Bath Ankylosing Spondylitis Functional Index; CRP: C-reactive protein; HADS: Hospital Anxiety and Depression Scale; IBD: inflammatory bowel disease.

**Figure 1.**
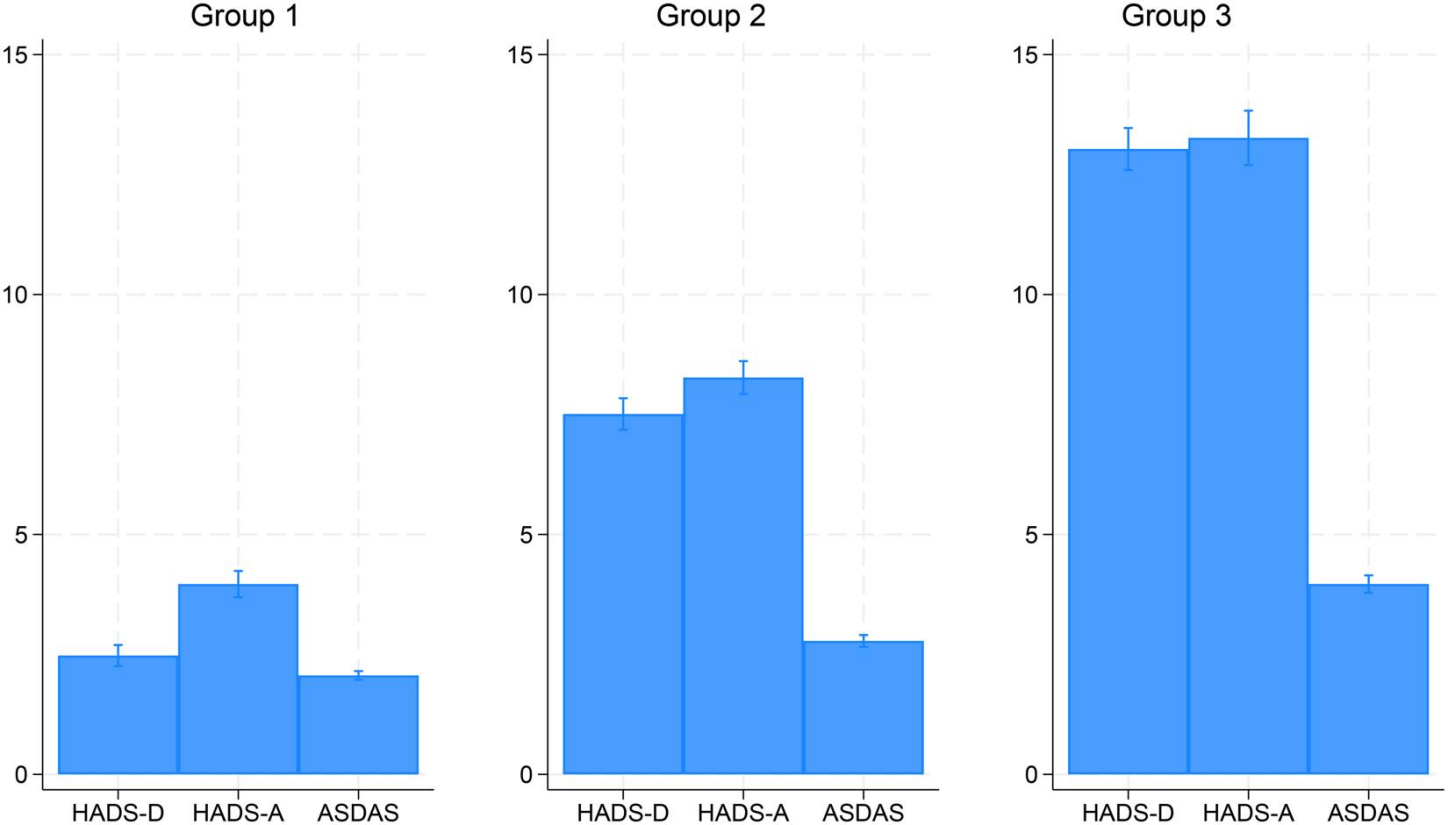
Latent class analysis showing concordant baseline values of three standardized indices: HADS-D, HADS-A and ASDAS. ASDAS: Axial Spondyloarthritis Disease Activity Score; HADS: Hospital Anxiety and Depression Scale subscores for depression (D) and anxiety (A)

The analysis identified three as the optimum number of latent classes (BIC/AIC shown in Supplementary Fig. S1). HADS subscores and ASDAS were generally concordant across groups; that is, group 1 had low scores (HADS-D 2.4, HADS-A 3.9, ASDAS2.1), group 2 medium scores (7.6, 8.3, 2.8, respectively) and group 3 high scores (13.2, 13.4, 4.0, respectively) (Fig. 1). There were no differences in age, sex, symptom or disease duration across latent groups (Table 1). However, the high-score group 3 generally had a higher proportion of patients with current peripheral (i.e. arthritis, dactylitis, enthesitis) involvement, higher median CRP and a lower proportion that were HLA-B27 positive (Table 1). From groups 1–3 (i.e. with increasing scores), educational attainment and perceived treatment helpfulness both decreased.

The number of latent groups and the concordance between HADS and ASDAS remained consistent across all sensitivity analyses (Supplementary Figs S2–S4); notably, analyses that included fatigue, BASFI and pain also demonstrated concordance between these indices and both ASDAS and HADS.

For the latent trajectory analysis within the subgroup of patients that underwent treatment change, 206 participants (mean age 37 years, 65% male) with longitudinal data were included, of whom 73 (35%) started NSAIDs, 23 (11%) csDMARD and 109 (53%) TNFi. In total, 62% were classified as radiographic axSpA and 80% were HLA-B27 positive. The mean disease duration was 6.0 (SD 7.9) years, mean baseline HADS-D 6.9 (SD 4.2), HADS-A 7.8 (4.4), ASDAS 3.3 (1.0); that is, higher than the overall cross-sectional study population (Table 2).

**Table 2.** Baseline characteristics of individuals included in the latent trajectory analysis over time and after treatment change

		All	Group 1	Group 2	Group 3	Group 4	*P*-value
N		206	112	14	69	11	
Age (years)		37.0 (11.8)	33.8 (10.8)	35.1 (11.8)	41.4 (11.4)	44.4 (12.7)	<0.001
Male		134 (65%)	76 (68%)	11 (79%)	40 (58%)	7 (64%)	0.38
Highest education attainment	Primary	14 (7%)	5 (4%)	2 (14%)	6 (9%)	1 (9%)	0.20
	Secondary	98 (48%)	48 (43%)	5 (36%)	40 (58%)	5 (45%)	
	University	94 (46%)	59 (53%)	7 (50%)	23 (33%)	5 (45%)	
Symptom duration (years)		11.5 (9.7)	10.6 (8.9)	9.1 (13.7)	13.0 (10.2)	15.2 (8.6)	0.17
Disease duration (years)		6.0 (7.9)	5.4 (7.0)	6.3 (11.7)	6.5 (8.4)	8.5 (7.2)	0.57
HLA-B27 positive		141 (80%)	86 (85%)	8 (62%)	39 (74%)	8 (80%)	0.13
Radiographic axSpA		128 (62%)	65 (58%)	9 (64%)	46 (67%)	8 (73%)	0.58
Peripheral arthritis		86 (43%)	44 (40%)	4 (31%)	32 (47%)	6 (55%)	0.52
Dactylitis		41 (20%)	21 (19%)	1 (8%)	15 (22%)	4 (36%)	0.35
Peripheral enthesitis		92 (45%)	46 (42%)	5 (38%)	34 (49%)	7 (64%)	0.44
Uveitis		39 (19%)	21 (19%)	4 (29%)	12 (18%)	2 (18%)	0.82
Psoriasis		18 (9%)	9 (8%)	0 (0%)	8 (12%)	1 (9%)	0.55
IBD		14 (7%)	7 (6%)	0 (0%)	6 (9%)	1 (10%)	0.67
CRP (mg/L)		10.0 (2.0, 19.4)	7.0 (2.0, 16.6)	15.3 (11.0, 32.7)	9.1 (2.8, 24.3)	32.2 (19.0, 53.3)	0.004
Perceived treatment helpfulness (0–10)		7.2 (2.2)	7.3 (2.0)	8.3 (2.1)	7.1 (2.3)	6.4 (2.7)	0.16
Baseline ASDAS		3.3 (1.0)	2.9 (0.8)	4.3 (1.1)	3.7 (1.0)	4.6 (0.6)	<0.001
Baseline HADS-depression (0–21)		7.8 (4.4)	4.8 (2.4)	11.5 (3.4)	10.5 (3.0)	15.7 (3.4)	<0.001
Baseline HADS-anxiety (0–21)		6.9 (4.2)	4.2 (2.7)	12.3 (2.2)	8.9 (2.9)	14.4 (2.7)	<0.001
Mean change ASDAS		−1.3 (1.1)	−1.2 (0.9)	−2.8 (1.1)	−1.3 (1.1)	−1.4 (1.1)	<0.001
Mean change HADS-D (0–21)		−2.3 (4.0)	−1.8 (2.9)	−11.4 (2.6)	−1.2 (3.1)	−3.2 (5.1)	<0.001
Mean change HADS-A (0–21)		−1.9 (3.6)	−1.4 (2.4)	−8.7 (4.5)	−1.6 (3.2)	−0.6 (5.3)	<0.001
NSAIDs initiators		73 (35%)	42 (38%)	3 (21%)	26 (38%)	2 (18%)	0.45
TNFi initiators		109 (53%)	52 (46%)	11 (79%)	37 (54%)	9 (82%)	0.026
csDMARD initiators		23 (11%)	17 (15%)	0 (0%)	6 (9%)	0 (0%)	0.15
Baseline latent Group 1		75 (36%)	74 (66%)	0 (0%)	1 (1%)	0 (0%)	<0.001
Baseline latent Group 2		94 (46%)	38 (34%)	4 (29%)	51 (74%)	1 (9%)	
Baseline latent Group 3		37 (18%)	0 (0%)	10 (71%)	17 (25%)	10 (91%)	

Data shown as *n* (%), mean (standard deviation) or median (interquartile range). Group 1: low baseline ASDAS/HADS; Group 2: high baseline with marked improvement; Group 3: moderate baseline; group 4: high baseline with limited improvement. ASAS-HI: Assessment of Spondyloarthritis International Society Health Index score; ASDAS: Axial Spondyloarthritis Disease Activity Score; BASDAI: Bath Ankylosing Spondylitis Disease Activity Index; BASFI: Bath Ankylosing Spondylitis Functional Index; CRP: C-reactive Protein; HADS: Hospital Anxiety and Depression Scale; IBD: Inflammatory Bowel Disease.

**Figure 2.**
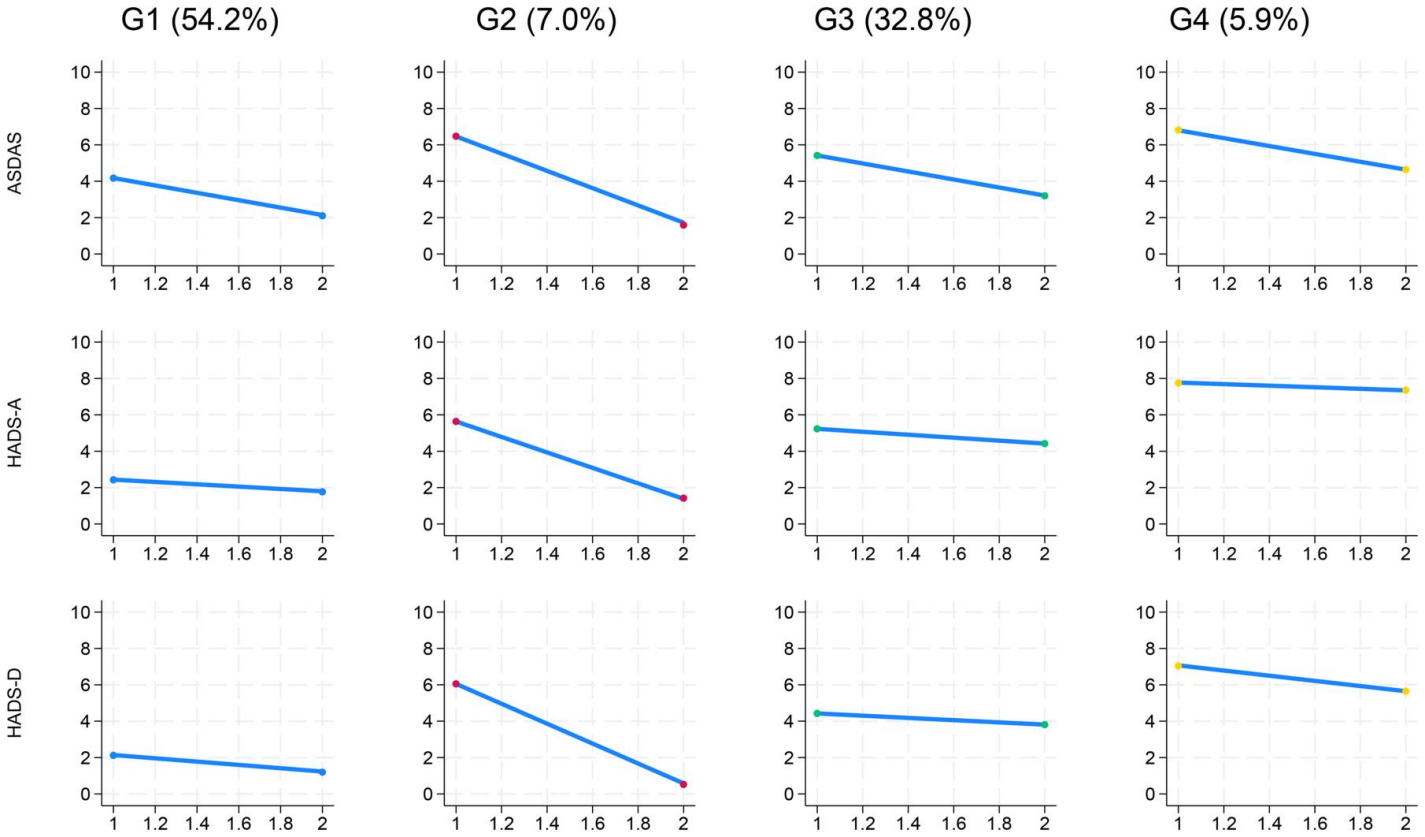
Latent trajectory groups showing concordant baseline and change in the three standardized indices: HADS-D, HADS-A and ASDAS. Points on the *x*-axis indicate baseline and reassessment (at 12–24 weeks). ASDAS: Axial Spondyloarthritis Disease Activity Score; HADS: Hospital Anxiety and Depression Scale subscores for depression (D) and anxiety (A)

The optimum number of trajectory groups was four (Supplementary Fig. S5, Fig. 2), across which the three indices (ASDAS, HADS-A, HADS-D) changed concordantly. Group 1 was the largest (54%), then G3 (33%), G2 (7%) and G4 (6%). Most patients in G1 corresponded to the baseline latent Group 1 (low score), G2 and 4 predominantly comprised patients from baseline Group 3 (high score), and G3 mostly included patients from baseline Group 2 (Table 2).

Group descriptions are relative: G1 started with lower scores for all three indices (ASDAS 2.9, HADS-D 4.8, HADS-A 4.2), G3 moderate (3.7, 10.5, 8.9, respectively), while G2 (4.3, 11.5, 12.3) and G4 (4.6, 15.7, 14.4) were higher. G1 and G3 had the lowest CRP and lowest proportion starting TNFi, while the opposite was true for G2 and G4. Mean improvements in ASDAS were numerically similar across G1, G3, G4 (reduction of 1.2–1.4 units), while G4 had minimum change in HASD-A (Table 2). G2 had the largest improvement across all three indices. Patient and disease characteristics were similar across all four groups, except mean age, which increased from G1 to G4. G3 and 4 trended towards having lower educational attainment and perceived treatment helpfulness, although not statistically significant. Sensitivity analysis additionally including fatigue, spinal pain and BASFI did not change the concordance of ASDAS and HADS scores (Supplementary Fig. S6).

## Discussion

In this study, we hypothesized that distinct groups of patients with axSpA might show discordance between disease activity and mental health symptoms; for example, individuals with a high burden of anxiety or depression despite low inflammatory disease activity. However, our findings showed that ASDAS scores and symptoms of anxiety and depression were closely aligned at baseline and tended to change in parallel following treatment change. In contrast to the baseline latent class analysis (three groups of low, medium and high ASDAS and HADS), the group with high-baseline scores in the latent trajectory analysis (characterized by higher CRP and proportion of TNFi starters) further separated into a group with good response and another group with blunted ASDAS and minimal HADS-A response.

There are undoubtedly patients who have high non-inflammatory symptom burden despite low inflammatory disease activity and vice versa. The fact that these patients did not emerge as a separate group in the current study could be due to two reasons. First, HADS and ASDAS are mostly subjective measures that may not sufficiently distinguish inflammatory and non-inflammatory symptoms. For example, severe inflammatory back pain will likely influence responses to ‘I feel if I am slowed down’ or ‘I can sit at ease and feel relaxed’ in HADS. Conversely, unmanaged depressive symptoms will influence how individuals respond to patient global of ASDAS. Even raised CRP could be confounded by BMI, which is associated with inflammatory and non-inflammatory symptoms [13]. Second, if the number of individuals with discordant ASDAS and HADS is limited or if the absolute differences between the standardized indices are small, then they may not appear as latent classes.

Our finding that ASDAS and HADS changed in tandem after treatment change is consistent with existing literature. For example, a *post hoc* analysis of the ASSERT trial showed that infliximab improved both axSpA and depressive symptom severity [14]. More pronounced reductions in inflammatory disease activity (by TNFi more than NSAIDs [15] or csDMARDs [16]) are generally associated with greater improvements in depressive symptoms. Similar associations are observed in the general population [17], particularly among those with elevated CRP [18], and are supported by mechanistic evidence from rheumatoid arthritis showing that proinflammatory cytokines can influence mood-regulating pathways and that immunomodulatory treatments may improve depressive symptoms independently of physical outcomes [19]. In ASSERT, however, the effect of infliximab on depressive symptoms was largely explained by improvements in self-reported axSpA symptoms, while no independent role for (CRP-mediated) inflammation was observed [14]. Future research should disentangle the mechanistic origins of depressive symptoms; for example, neuro-biochemical depression resulting from systemic inflammation *vs* depressive symptoms secondary to pain and/or other axSpA symptoms. Longitudinal cohort studies with repeated measures of inflammation and mental health might help clarify whether inflammatory activity precedes and contributes to depressive symptoms, or whether depression amplifies the perception and reporting of axSpA disease activity. Neuroimaging and biomarker studies in axSpA may also help illuminate shared biological pathways linking inflammation and depression. Finally, we also observed that the high-symptom group had lower educational attainment. Although our analyses were not designed to assess predictors of class membership, education may reflect broader socioeconomic influences that can shape both mental health and reported disease burden, an area that warrants further investigation.

While the prevalence of depressive symptoms is high in axSpA [2, 20], this risk is not unique to axSpA but extends across spondyloarthritis, with an even greater burden reported in psoriatic arthritis [21]. The main clinical implication of the current findings is that measurable mental health symptoms generally improve as inflammatory axSpA disease activity after treatment initiation. Whether patients benefit from knowing that mental health symptoms and axSpA disease activity jointly improve when starting treatment remains to be shown and should be a focus of future research.

A key strength of this study is the large, international sample, which enhances the generalizability of our findings. In addition, the use of data-driven latent class and trajectory modelling enabled exploration of underlying symptom patterns without prespecified assumptions. Our study has several limitations. First, HADS was created to measure anxiety and depressive symptoms in a general medical population; although extensively used in rheumatic and musculoskeletal diseases [22, 23], its performance in these populations remains unclear. Moreover, HADS is primarily used for screening and does not provide gold standard diagnosis. Second, the number of patients with longitudinal data was relatively low and may be underpowered to detect smaller latent trajectory groups. Longer follow-up may reveal differences in trajectories that are missed within the limited follow-up. Third, different treatment groups (NSAIDs, csDMARD, TNFi) were pooled in the trajectory analysis by necessity in the interest of statistical power but may potentially mask smaller effects. For example, Groups 2 and 4 in the latent trajectory analysis had higher proportion of TNFi initiators and the highest starting ASDAS and HADS scores, thus the effect of starting severity and treatment modality is difficult to untangle. Lastly, although the ASAS HI validation study was a large international study, it was designed for an alternate hypothesis and aspects of its design (e.g. specifying the proportion radiographic axSpA and exclusion of individuals with severe comorbidities) may make the current results less generalizable to other axSpA populations.

In conclusion, results of this study show that ASDAS and anxiety and depressive symptoms are closely related and change concordantly with treatment, suggesting that musculoskeletal and mental health symptoms influence each other. Whether axSpA patients benefit from knowing that mental health symptoms generally improve jointly with disease activity when starting treatment remains to be shown. Future research should seek to better disentangle the mechanistic origins of depressive symptoms in axSpA and assess evolution of mental health symptoms over long-term follow-up.

## Supplementary material

Supplementary material is available at *Rheumatology* online.

## Supplementary Material

keaf506_Supplementary_Data

## Data Availability

Data from the ASAS HI Validation Study are available from the senior authors upon reasonable request.
