# Supplementary material for: Disease activity and mental health symptoms in axial spondyloarthritis: concordant or discordant?
Source: Rheumatology (Oxford). 2025 Sep 29;65(1):keaf506. doi: 10.1093/rheumatology/keaf506 (PMC12862361; doi:10.1093/rheumatology/keaf506)
Supplement: keaf506_Supplementary_Data [file keaf506_supplementary_data.docx]

**Disease activity and mental health symptoms in axial spondyloarthritis: concordant or discordant?**

Sizheng Steven Zhao^1,2^, Casper Webers^3^, Elena Nikiphorou^4,5,6^, Désirée van der Heijde^7^, Jurgen Braun^8^, Uta Kiltz^8^, Sofia Ramiro^9 #^, Annelies Boonen^3,10 #^

#Contributed equally

1 Centre for Musculoskeletal Research, Division of Musculoskeletal and Dermatological Science, University of Manchester, Manchester, UK.

2. NIHR Manchester Biomedical Research Centre, Manchester University NHS Foundation Trust, Manchester

3 Care and Public Health Research Institute (Caphri), Maastricht University, Maastricht, the Netherlands.

4 School of Inflammation and Microbial Sciences, Centre for Rheumatic Diseases, King’s College London, London, UK.

5 Centre for Education, King’s College London, London, UK.

6 Rheumatology Department, King’s College Hospital NHS Foundation Trust, London, UK.

7 Leiden University Medical Center, Leiden, the Netherlands.

8 Rheumazentrum Ruhrgebiet, Herne, Ruhr-University Bochum, Germany.

9 Leiden University Medical Center, Leiden and Zuyderland Medical Center, Heerlen, the Netherlands.

10 Maastricht UMC+, Dept of Rheumatology, Maastricht, the Netherlands.

Correspondence to: Dr Sizheng S Zhao. Centre for Musculoskeletal Research, Division of Musculoskeletal and Dermatological Science, School of Biological Sciences, Faculty of Biological Medicine and Health, The University of Manchester, Manchester Academic Health Science Centre, Oxford Road, Manchester, M13 9LJ, UK. Email: Sizheng.zhao@manchester.ac.uk

Contents

[Supplementary Figure S1. Model fit statistics showing three as the optimum number of latent classes for the baseline latent class analysis 3](#_Toc210903699)

[Supplementary Figure S2. Baseline latent class sensitivity analysis without standardizing indices. 3](#_Toc210903700)

[Supplementary Figure S3. Baseline latent class sensitivity analysis including additional symptom indicators. 4](#_Toc210903701)

[Supplementary Figure S4. Baseline latent class sensitivity analysis among only the longitudinal “responsiveness” arm. 4](#_Toc210903702)

[Supplementary Figure S5. Model fit statistics showing four as the optimum number of groups for the longitudinal trajectory analysis 5](#_Toc210903703)

[Supplementary Figure S6. Latent trajectory sensitivity analysis additionally including BASFI, pain and fatigue. 6](#_Toc210903704)

# Supplementary Figure S1. Model fit statistics showing three as the optimum number of latent classes for the baseline latent class analysis

C: number of classes (e.g., c2 denotes model with 2 classes). AIC: Akaike Information Criterion, BIC: Bayesian Information Criterion.

# Supplementary Figure S2. Baseline latent class sensitivity analysis without standardizing indices.


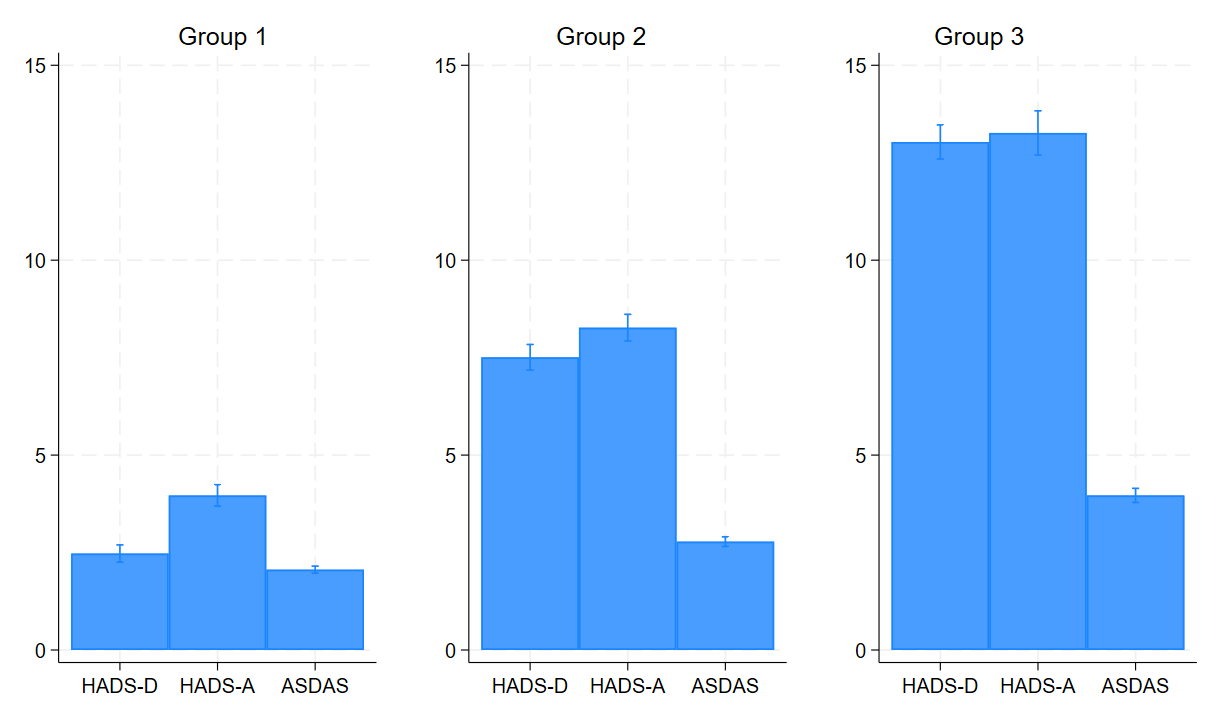


HADS: Hospital Anxiety and Depression Scale; ASDAS: Axial Spondyloarthritis Disease Activity Score

# Supplementary Figure S3. Baseline latent class sensitivity analysis including additional symptom indicators.


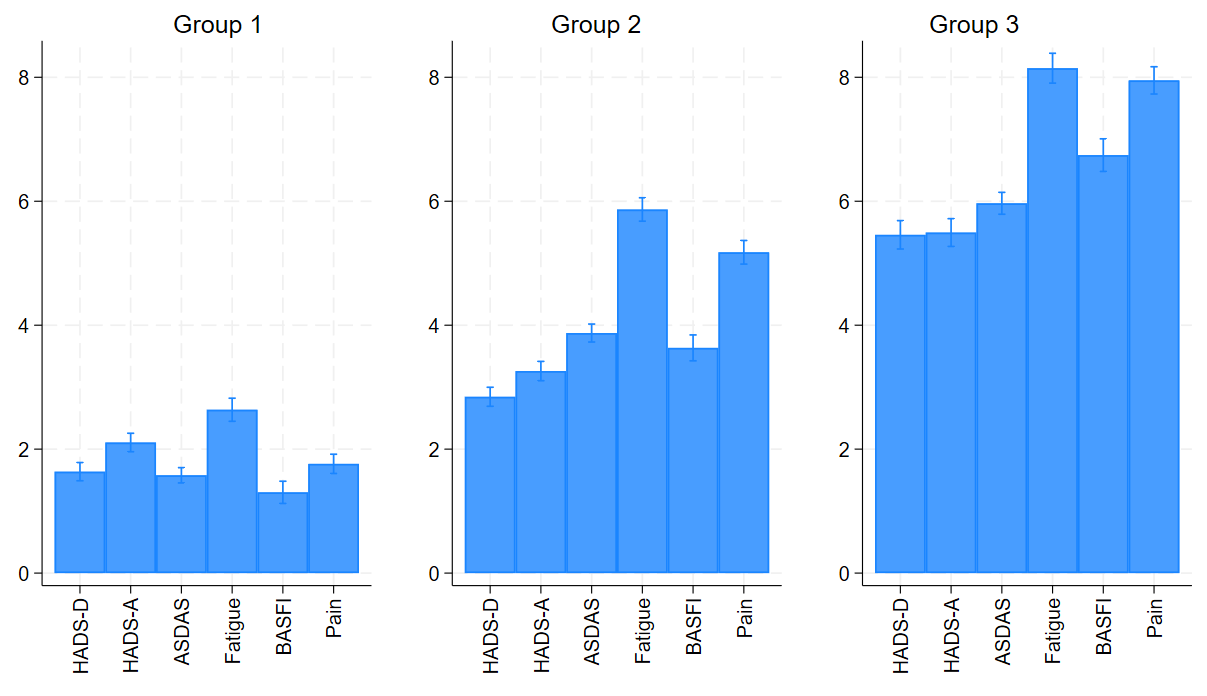


HADS: Hospital Anxiety and Depression Scale; ASDAS: Axial Spondyloarthritis Disease Activity Score; BASDAI question on fatigue; BASFI: Bath Ankylosing Spondylitis Functional Index.

# Supplementary Figure S4. Baseline latent class sensitivity analysis among only the longitudinal “responsiveness” arm.


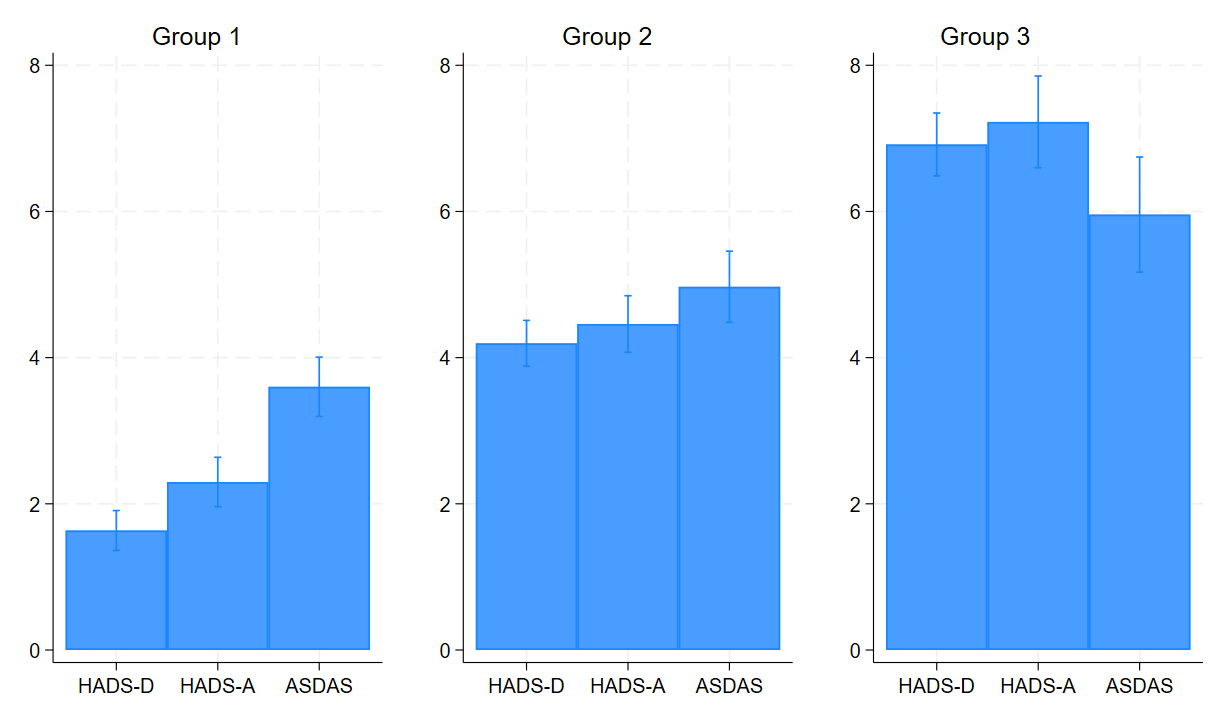


HADS: Hospital Anxiety and Depression Scale; ASDAS: Axial Spondyloarthritis Disease Activity Score

# Supplementary Figure S5. Model fit statistics showing four as the optimum number of groups for the longitudinal trajectory analysis

C: number of classes (e.g., c2 denotes model with 2 classes). AIC: Akaike Information Criterion, BIC: Bayesian Information Criterion.

# Supplementary Figure S6. Latent trajectory sensitivity analysis additionally including BASFI, pain and fatigue.


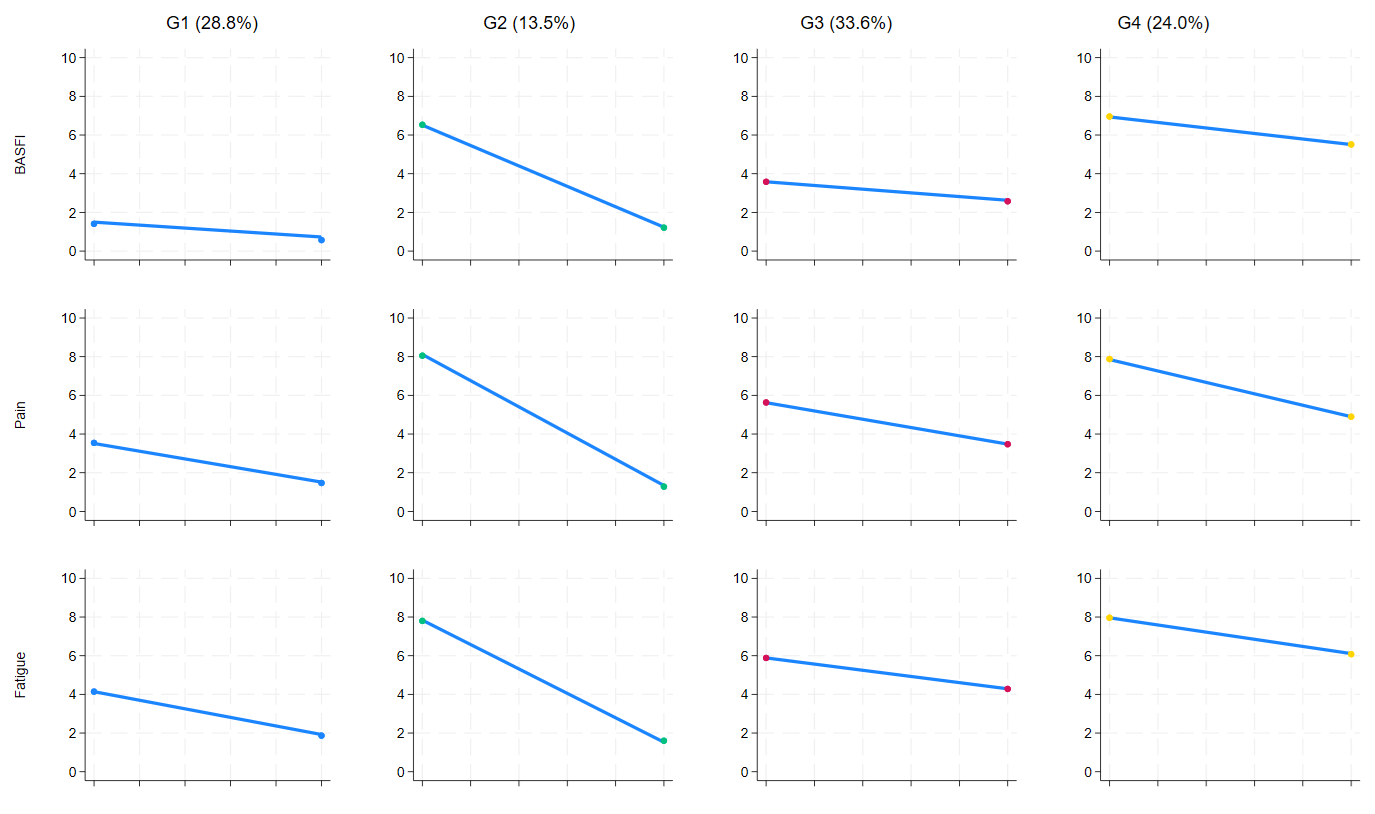


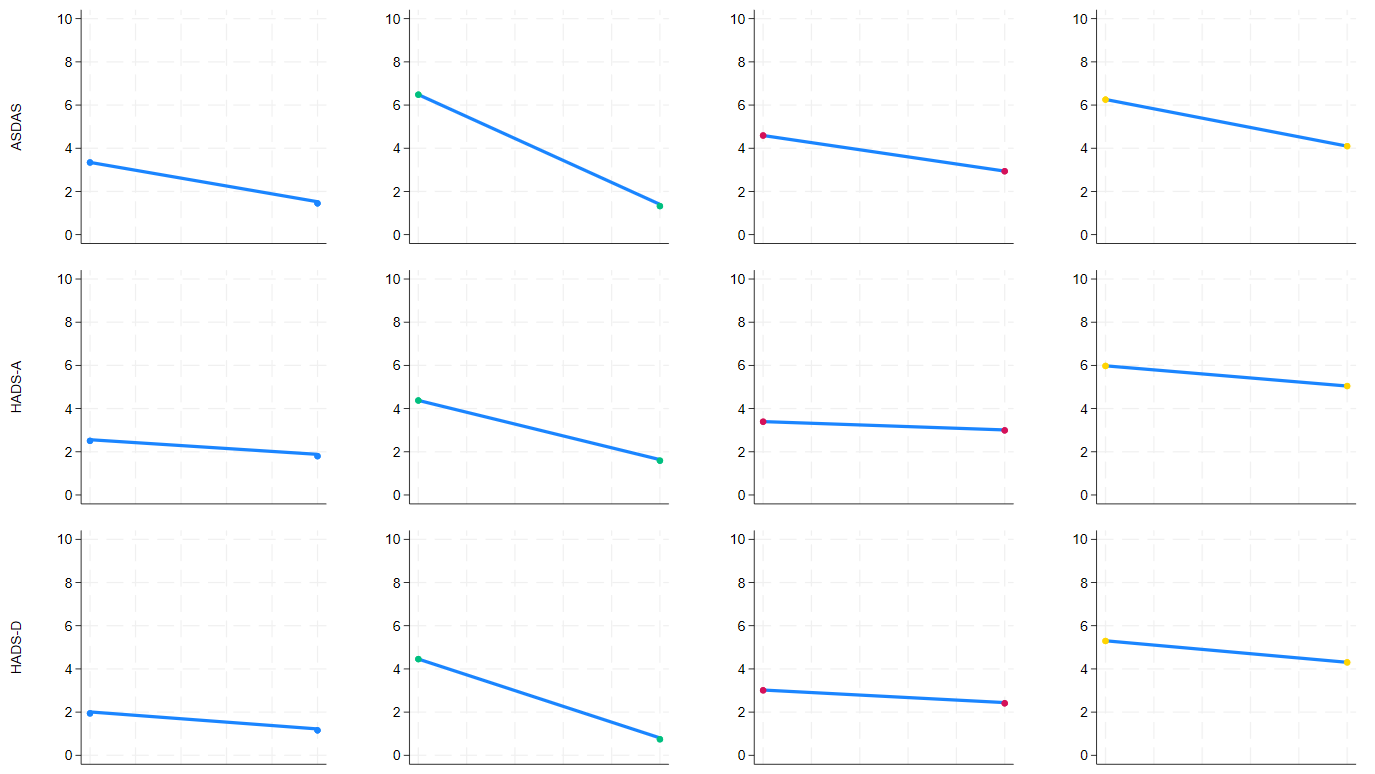


HADS: Hospital Anxiety and Depression Scale; ASDAS: Axial Spondyloarthritis Disease Activity Score; BASDAI question on fatigue; BASFI: Bath Ankylosing Spondylitis Functional Index.
